# Effects of bariatric surgery on renal function: a retrospective cohort study comparing one-year outcomes between one-anastomosis gastric bypass and Roux-en-Y gastric bypass

**DOI:** 10.1590/1516-3180.2023.0161.R1.08022024

**Published:** 2024-05-31

**Authors:** Victor Kenzo Ivano, Marcelo Hatto, Fernanda Teramoto, Paolla Ravida Alves de Macedo, Martinho Antonio Gestic, Murillo Pimentel Utrini, Felipe David Mendonça Chaim, Almino Cardoso Ramos, Francisco Callejas-Neto, Elinton Adami Chaim, Everton Cazzo

**Affiliations:** I MD. Postgraduate Student, Department of Surgery, School of Medical Sciences, Universidade Estadual de Campinas (UNICAMP), Campinas (SP), Brazil.; II MD. Postgraduate Student, Department of Surgery, School of Medical Sciences, Universidade Estadual de Campinas (UNICAMP), Campinas (SP), Brazil.; III MD. Medical Resident, Department of Surgery, School of Medical Sciences, Universidade Estadual de Campinas (UNICAMP), Campinas (SP), Brazil.; IV MD. Medical Resident, Department of Surgery School of Medical Sciences, Universidade Estadual de Campinas (UNICAMP), Campinas (SP), Brazil.; V MD, MSc. Assistant lecturer, Department of Surgery, School of Medical Sciences, Universidade State University of Campinas (UNICAMP), Campinas (SP), Brazil.; VI MD. Assistant Lecturer, Department of Surgery, School of Medical Sciences, Universidade Estadual de Campinas (UNICAMP), Campinas (SP), Brazil.; VII MD, PhD. Assistant Lecturer, Department of Surgery, School of Medical Sciences, Universidade Estadual de Campinas (UNICAMP), Campinas (SP), Brazil.; VIII MD, PhD. Department of Surgery, School of Medical Sciences, Universidade Estadual de Campinas (UNICAMP), Campinas (SP), Brazil.; IX MD, MSc. Assistant Professor, Department of Surgery, School of Medical Sciences, Universidade Estadual de Campinas (UNICAMP), Campinas (SP), Brazil.; X MD, PhD. Full Professor, Department of Surgery, School of Medical Sciences, Universidade Estadual de Campinas (UNICAMP), Campinas (SP), Brazil.; XI MD, PhD. Associate Professor, Department of Surgery, School of Medical Sciences, Universidade Estadual de Campinas (UNICAMP), Campinas (SP), Brazil.

**Keywords:** Gastric bypass, Bariatric surgery, Glomerular filtration rate, Kidney diseases, Obesity, One-anastomosis gastric bypass, Roux-en-Y gastric bypass, Renal function

## Abstract

**BACKGROUND:**

Evidence on the effect of one-anastomosis gastric bypass (OAGB) on renal function is limited.

**OBJECTIVE:**

To compare the evolution of estimated renal function observed 1 year after OAGB and Roux-en-Y gastric bypass (RYGB) in individuals with obesity.

**DESIGN AND SETTING:**

Observational, analytical, and retrospective cohort study. Tertiary-level university hospital.

**METHODS:**

This study used a prospectively collected database of individuals who consecutively underwent bariatric surgery. Renal function was assessed by calculating the estimated glomerular filtration rate (eGFR), according to the Chronic Kidney Disease Epidemiology Collaboration. The one-year variation in the eGFR was compared between the procedures.

**RESULTS:**

No significant differences in age, sex, obesity-associated conditions, or body mass index were observed among individuals who underwent either OAGB or RYGB. OAGB led to a significantly higher percentage of total (P = 0.007) and excess weight loss (P = 0.026). Both OAGB and RYGB led to significantly higher values of eGFR (103.9 ± 22 *versus* 116.1 ± 13.3; P = 0.007, and 102.4 ± 19 *versus* 113.2 ± 13.3; P < 0.001, respectively). The one-year variation in eGFR was 11 ± 16.2% after OAGB and 16.7 ± 26.3% after RYGB (P = 0.3). Younger age and lower baseline eGFR were independently associated with greater postoperative improvement in renal function (P < 0.001).

**CONCLUSION:**

Compared with RYGB, OAGB led to an equivalent improvement in renal function 1 year after the procedure, along with greater weight loss.

## INTRODUCTION

In the recent decades, obesity has reached worrisome epidemic proportions worldwide, compromising the life expectancy and quality of life of affected individuals. According to World Health Organization estimates, nearly 3 million deaths each year are directly attributable to obesity, mainly because of major cardiovascular events.^1^ Obesity and its related conditions are also significantly associated with impairment of renal function and the development of end-stage chronic kidney disease (CKD). Several key pathophysiological factors are seemingly involved in this association, such as insulin resistance, diabetes, hypertension, accumulation of visceral fat, chronic inflammation, and hyperuricemia.^2^ Evidence has also been reported, demonstrating that obesity acts as an independent risk factor for progression to CKD, both indirectly through diabetes and hypertension, as well as through a so-called obesity-related glomerulopathy (ORG), which is pathologically defined as glomerulomegaly and segmental focal glomerulosclerosis occurring in individuals with obesity regardless of other obesity-related medical conditions.^3,4^ Although the pathophysiology of ORG remains unclear, obesity may initially induce hyperfiltration and increases tubular sodium reabsorption, resulting in glomerular hypertension and activation of the renin–angiotensin–aldosterone system, associated with inflammation and imbalance of adipokines. The clinical course is characterized by stable or slowly progressing proteinuria, and up to one-third of patients develop renal failure and end-stage CKD.^5-7^

Weight loss interventions are effective in mitigating or even resolving ORG.^8^ Considering that bariatric surgery (BS) is the most effective method that leads to long-term significant and sustained weight loss in individuals with refractory obesity, it also reportedly improves long-term kidney function in individuals with obesity. Several studies have demonstrated the beneficial effects of Roux-en-Y gastric bypass (RYGB) on renal function.^9,10^ Garcia et al.^11^ analyzed individuals who underwent RYGB and observed significant improvement in the estimated glomerular filtration rate (eGFR) 1 year postoperatively. Moreover, evidence that improvement of renal function after BS may occur regardless of weight loss or glycemic control has been reported, thus corroborating the hypothesis that adipokine homeostasis, enterohormonal mechanisms, and reduction of systemic inflammation may play pivotal roles in post-BS nephroprotection.^12,13^

One-anastomosis gastric bypass (OAGB) has emerged more recently as a promising and highly effective operation to treat obesity, with reports indicating both weight loss and resolution rates of diabetes as superior to those observed after RYGB.^14,15^ OAGB is based on a simplification of RYGB, with a single anastomosis (gastroenterostomy) and no enteroenterostomy, which is generally associated with a reduction in technical complexity and significantly lower operative times.^16^ However, to date and to the best of our knowledge, data reporting the impact of OAGB on renal function are scarce. In a single study, Bassiony et al. evaluated creatinine clearance in 10 patients undergoing OAGB and 47 patients undergoing sleeve gastrectomy, demonstrating a significant reduction in glomerular hyperfiltration and urinary protein excretion 6 months after both operations, without significant difference between the techniques.^17^

## OBJECTIVE

This study aimed to compare the evolution of estimated renal function observed 1 year after OAGB and RYGB in individuals with obesity.

## METHODS

### Study Design

This observational, analytical, and retrospective study was based on a prospectively collected database of individuals who consecutively underwent BS at a tertiary-level university hospital between 2018 and 2019. BS was performed during the implementation of OAGB at this facility when individuals underwent either OAGB or RYGB without pre-established differences in the indications for both operations. OAGB was performed on days when the entire research team responsible for the trial was identified at http://ensaiosclinicos.gov.br as RBR-59k78k was present; RYGB was performed in the remaining cases. The research team was available monthly.

The main outcome considered was the variation in renal function 1 year postoperatively, which was compared between the RYGB and OAGB groups.

The study was approved by the Ethical Committee of the Universidade Estadual de Campinas under reference number CAAE 55545422.9.0000.5404 on March 25, 2022. All participants signed an informed consent form. All procedures involving human participants performed in this study were in accordance with the ethical standards of the institutional and/or national research committee and with the 1964 Helsinki Declaration and its later amendments or comparable ethical standards. Informed consent was obtained from all the participants.

### Study Population

We included individuals aged 18–65 years of any sex who underwent either OAGB or RYGB between 2018 and 2019. Individuals with incomplete medical records, belonging to vulnerable groups (minors or with mental or intellectual disabilities), or who did not consent to study participation were excluded. Surgery was indicated according to the National Institutes of Health Consensus criteria (body mass index [BMI] ≥ 40 kg/m^2^ or BMI ≥ 35 kg/m^2^ with obesity-related medical conditions).

No specific criteria were established for the participants to undergo either OAGB or RYGB, except in situations where there were contraindications for OAGB (severe gastroesophageal reflux, preoperative esophagogastric intestinal metaplasia, or an antecedent of familial gastric cancer). All patients underwent consecutive operations and were informed of the technique adopted prior to the procedure. OAGB was performed on days when the entire research team gathered, whereas RYGB was performed on the remaining days. The selected patients for surgery followed a regular hospital schedule.

### Surgical Techniques

#### OAGB

**Figure 1:**
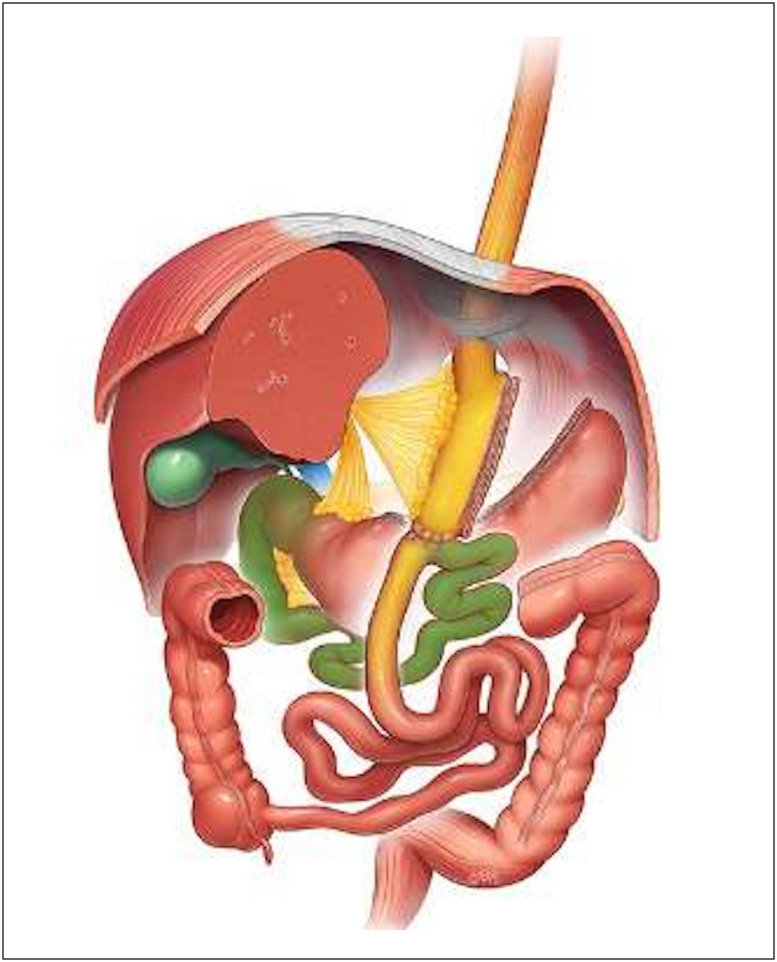
Graphic representation of one anastomosis gastric bypass. Source: © Dr Levent Efe, courtesy of IFSO.^48^

The main features of OAGB include approximately 15 cm gastric pouch alongside a 200 cm biliopancreatic limb and a common channel comprising the remainder of the small intestine. Figure 1 presents a graphical representation of the surgical technique.

#### RYGB

**Figure 2:**
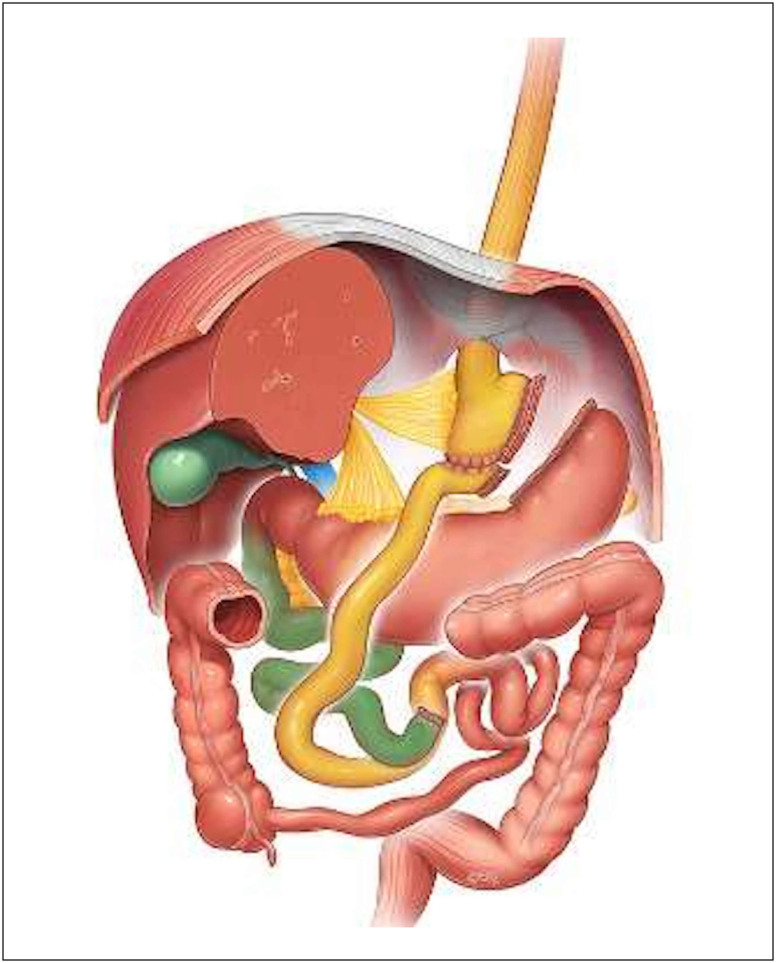
Graphic representation of Roux-en-Y gastric bypass. Source: © Dr Levent Efe, courtesy of IFSO.^48^

The main features of RYGB include an approximately 30-mL gastric pouch, 100-cm biliopancreatic loop, 150-cm alimentary limb, and a common channel comprising the remainder of the small intestine. Figure 2 presents a graphical representation of the surgical technique.

### Study Variables

#### Demographic, Clinical, Anthropometric, and Biochemical Variables

The following variables were considered: age at surgery, sex, weight, BMI, and presence of obesity-associated medical conditions. Weight loss was analyzed as a percentage of total weight loss (%TWL) and excess weight loss (%EWL). Pre- and postoperative fasting glucose, serum urea, creatinine, and albumin levels were assessed. Percentage variations in these biochemical variables, considering their pre- and postoperative values, were calculated.

#### Renal Function Assessment

Renal function was assessed using eGFR, which was calculated using the Chronic Kidney Disease Epidemiology Collaboration formula. The percentage variation in the CKD-EPI was calculated 1 year postoperatively.

The CKD-EPI formula was calculated according to the proposition of Levey et al.^18^ It was used to evaluate the eGFR and has the advantage of not considering the patient's weight since, in individuals with obesity, the formulas that consider this variable tend to overestimate the true values of GFR.^19^ The CKD-EPI formula is expressed as a single equation as follows:


$$\begin{array}{c} \text{eGFR}=141\times \min(\text{Scr}/\kappa ,1{)}^{\text{a}}\times \max(\text{Scr}/\kappa ,1{)}^{-1.209}\times \\ 0.993^{\text{Age}}\times 1.018[\text{iffemale}]\times \text{1}.\text{159}[\text{ifBlack}] \end{array}$$

Scr is serum creatinine (mg/dL), κ is 0.7 for women and 0.9 for men, α is −0.329 for women and −0.411 for men, min indicates the minimum of Scr/κ or 1, and max indicates the maximum of Scr/κ or 1.

### Statistical Analysis

Proportions were compared using the chi-square test or Fisher's exact test, when necessary. Normality was assessed using the Shapiro–Wilk test. Comparisons of continuous or ordinal measurements between the two assessments were performed using the Mann–Whitney U test. To assess the associations of the study variables with the main outcome (one-year variation in GFR), simple and multiple regression analyses were performed. The level of significance was set at 5% (P < 0.05).

## RESULTS

The average age of the study participants was 38.6 ± 9.1 years, and 87% were female. The mean preoperative BMI was 39 ± 5.8 kg/m^2^; postoperatively, it significantly decreased to 27.9 ± 4.3 kg/m^2^ (P < 0.001). Regarding obesity-related conditions, 43.2% presented with hypertension, and 26% had type 2 diabetes. Overall, the participants experienced a %TWL of 23.2 ± 11.3% and %EWL of 77.3 ± 36.7%.

No significant differences in age, sex, obesity-associated conditions, or BMI were observed among individuals who underwent either OAGB or RYGB. OAGB led to significantly higher %TWL (P = 0.007) and %EWL (P = 0.026) (Table 1).

**Table 1:** Comparison of baseline characteristics and postoperative outcomes between patients who underwent one anastomosis gastric bypass and those who underwent Roux-en-Y gastric bypass

		OAGB	RYGB	P value
N		46	100	NA
Age (years)		37.4 ± 8	39.2 ± 9.6	0.28
Sex		Male: 5 (10.9%) Female: 41 (89.1%)	Male: 14 (14%) Female: 86 (86%)	0.60
Preoperative BMI (kg/m^2^)		38.3 ± 5.4	37.3 ± 3.7	0.22
Postoperative BMI (kg/m^2^)		27 ± 3.9	28.4 ± 4.5	0.07
%TWL		26.9 ± 10.3%	21.4 ± 11.3%	0.007
%EWL		87.4 ± 30.7%	72.5 ± 38.5%	0.026
Preoperative glucose (mg/dL)		87.8 ± 14.7	91.6 ± 20.3	0.27
Postoperative glucose (mg/dL)		82 ± 8.5	81.8 ± 10	0.92
% Δ Glucose		−6.4 ± 15%	−6.9±15.5%	0.89
Preoperative creatinine (mg/dL)		0.8 ± 0.2	0.8 ± 0.2	0.99
Postoperative creatinine (mg/dL)		0.6 ± 0.1	0.6 ± 0.1	0.54
% Δ Creatinine		−14.6 ± 14.3%	−17 ± 20.5%	0.59
Preoperative urea (mg/dL)		24.7 ± 9.8	26.6 ± 9.8	0.29
Postoperative urea (mg/dL)		25.6 ± 6.8	25.1 ± 7	0.82
% Δ Urea		2.9 ± 20.9%	−4.7 ± 26.5%	0.39
Preoperative eGFR (mL/min/1.73m²)		103.9 ± 22	102.4 ± 19	0.69
Postoperative eGFR (mL/min/1.73m²)		116.1 ± 13.3	113.2 ± 13.3	0.33
% Δ eGFR		11 ± 16.2%	16.7 ± 26.3%	0.30
Preoperative obesity-associated conditions				
	Type 2 diabetes – N (%)	15 (32.6%)	23 (23%)	0.22
	Hypertension – N (%)	19 (41.3%)	44 (44%)	0.76
Postoperative obesity-associated conditions				
	Type 2 diabetes – N (%)	1 (2.2%)	6 (6%)	0.31
	Hypertension – N (%)	3 (6.5%)	9 (9%)	0.64
Diabetes remission rate (%)		93.3%	73.9%	0.13
Hypertension remission rate (%)		84.2%	79.5%	0.67

OAGB = one anastomosis gastric bypass; RYGB = Roux-en-Y gastric bypass; n = number of individuals; BMI = body mass index; eGFR = estimated glomerular filtration rate; % Δ = percentage of variation; %TWL = percentage of total weight loss; %EWL = percentage of excess weight loss.

**Bold** indicates statistical significance.

Regarding biochemical examinations, patients who underwent either RYGB or OAGB presented significantly decreased postoperative glucose, creatinine, hemoglobin, ferritin, and albumin levels. The urea, aminotransferase, and serum iron levels did not change significantly after either procedure. Table 2 presents the evolution of the biochemical parameters after both procedures.

**Table 2:** Biochemical changes 1 year after one anastomosis gastric bypass and Roux-en-Y gastric bypass

One-anastomosis gastric bypass (n = 46)			
	Preoperative	Postoperative	P value
BMI (kg/m^2^)	38.3 ± 5.4	27 ± 3.9	<0.001
Glucose (mg/dL)	87.8 ± 14.7	82 ± 8.5	0.047
Creatinine (mg/dL)	0.8 ± 0.2	0.6 ± 0.1	0.001
Urea (mg/dL)	24.7 ± 9.8	25.6 ± 6.8	0.75
eGFR (mL/min/1.73m²)	103.9 ± 22	116.1 ± 13.3	0.007
AST (IU/L)	23.2 ± 9.6	23.8 ± 11.3	0.82
ALT (IU/L)	30.6 ± 24.2	27.2 ± 15.2	0.53
Hemoglobin (g/dL)	13.9 ± 1.1	13 ± 1.1	0.002
Ferritin (μg/L)	185.9 ± 103.2	94.2 ± 112.4	0.03
Serum iron (μg/dL)	69.1 ± 16.1	89.5 ± 32.9	0.08
Albumin (g/dL)	4.3 ± 0.3	4.1 ± 0.3	0.01
Roux-em-Y gastric bypass (n = 100)			
BMI (kg/m^2^)	37.3 ± 3.7	28.4 ± 4.5	<0.001
Glucose (mg/dL)	91.6 ± 20.3	81.8 ± 10	0.002
Creatinine (mg/dL)	0.8 ± 0.2	0.6 ± 0.1	<0.0001
Urea (mg/dL)	26.6 ± 9.8	25.1 ± 7	0.39
eGFR (mL/min/1.73m²)	102.4 ± 19	113.2 ± 13.3	<0.0001
AST (IU/L)	21.8 ± 7.3	22.8 ± 19.9	0.64
ALT (IU/L)	26.7 ± 16.1	26.6 ± 40.9	0.99
Hemoglobin (g/dL)	13.9 ± 1.3	13.1 ± 1.3	0.01
Ferritin (μg/L)	233.9 ± 249.3	138.8 ± 149	0.01
Serum iron (μg/dL)	71.2 ± 28.7	83 ± 38.1	0.08
Albumin (g/dL)	4.3 ± 0.3	4.1 ± 0.3	<0.0001

n = number of individuals; BMI = body mass index; AST = aspartate aminotransferase; ALT = alanine aminotransferase; eGFR = estimated glomerular filtration rate.

**Bold** indicates statistical significance.

Considering the postoperative variation of renal function, both OAGB and RYGB led to significantly higher values of eGFR (103.9 ± 22 *versus* 116.1 ± 13.3; P = 0.007, and 102.4 ± 19 *versus* 113.2 ± 13.3; P < 0.001, respectively). The one-year variation of eGFR was 11 ± 16.2% after OAGB and 16.7 ± 26.3% after RYGB; no significant difference was observed between the two procedures (P = 0.3). The one-year postoperative variations in glucose, creatinine, and urea levels did not significantly differ between the procedures (Table 1).

In the univariate regression analysis enrolling the entire cohort, the main study outcome (one-year variation of GFR) was significantly associated with baseline creatinine (R = 0.80; P < 0.001) and baseline GFR (R = -0.85; P < 0.001); there was also a marginal association with age (R = 0.20; P = 0.07). Multivariate analysis was performed through multiple regression enrolling these three variables and showed that both age (R = −0.31; P < 0.001) and baseline GFR (R = -0.99; P < 0.001) were independently and negatively associated with the variation in GFR. Thus, the younger the age and the lower the GFR at surgery, the higher the postoperative increase in GFR. Table 3 summarizes the results of the simple and multiple regression analyses.

**Table 3:** Correlation analyses between the main study outcome (one-year variation of glomerular filtration rate) and study variables

Univariate analysis (simple regression)		
Variable	Regression coefficient	P value
*Age*	*0.20*	*0.07*
BL BMI	0.07	0.41
%TWL	0.18	0.12
%EWL	0.17	0.14
BL glucose	0.13	0.26
BL insulin	−0.35	0.81
BL creatinine	0.80	<0.001
BL urea	0.14	0.74
BL albumin	0.03	0.77
BL hemoglobin A1c	0.06	0.69
BL eGFR	−0.85	<0.001
Multivariate analysis (multiple regression)		
Age	−0.31	<0.001
BL creatinine	−0.16	0.25
BL eGFR	−0.99	<0.001

BL = baseline; BMI = body mass index; %TWL = percentage of total weight loss; %EWL = percentage of excess weight loss; eGFR = estimated glomerular filtration rate.

**Bold** indicates statistical significance. *Italic* indicates a marginal association.

## DISCUSSION

The current study demonstrated that both OAGB and RYGB promoted the recovery of renal function after 1 year. Both procedures demonstrated statistically comparable results in terms of the percentage variation in eGFR. Meanwhile, regarding weight, OAGB led to significantly greater weight loss than RYGB. Thus, although both procedures lead to equivalent benefits in relation to renal function, OAGB is more advantageous in terms of weight loss.

Several case series, retrospective and prospective studies, and systematic reviews have demonstrated post-BS improvement in renal function in patients with obesity, in addition to various other benefits in quality of life, metabolic control, blood pressure, and other conditions related to excess weight.^20-24^ Garcia et al.^11^ analyzed 109 patients who underwent RYGB and demonstrated a significant improvement in GFR 1 year postoperatively, which was more pronounced in younger individuals without hypofiltration. Interestingly, in this study, no significant correlation was identified between the improvement in kidney function and presence of obesity-associated conditions, such as diabetes and hypertension, or with greater loss of excess weight. This finding of renal improvement independent of the magnitude of weight loss was reinforced by a systematic review conducted by Scheurlen et al.,^12^ who enrolled 15 studies involving 2,145 patients undergoing RYGB and reported that patients had improved renal function regardless of weight loss or glycemic control.

However, the mechanisms underlying renal recovery after BS are unclear. They may be related to several different factors, which are seemingly linked, but far from restricted to, weight loss itself, as well as decreased visceral fat-associated inflammation, incretin activity on insulin sensitivity and pancreatic endocrine function, incretin natriuretic effect, improvement of hypertension, among others.^25^ Both OAGB and RYGB are reportedly capable of producing massive weight loss alongside significant metabolic improvement, which are likely to positively affect renal function, as observed in the current study. Regarding enterohormonal secretion, an interesting study by DeBandt et al. demonstrated no significant differences in the postprandial levels of glucagon-like peptide-1 (GLP-1), peptide YY, or ghrelin between OAGB and RYGB; however, glucose-dependent insulinotropic polypeptide levels tended to be lower with OAGB than with RYGB.^26^

The possibility of superior weight loss provided by OAGB compared to RYGB in the current study has been previously reported, although this remains debatable. Two pioneering studies comparing these techniques, the Y-OMEGA^27^ and Taiwan trial,^28^ demonstrated that both procedures led to similar weight loss, although OAGB promoted more metabolic improvement in relation to glucose metabolism and diabetes resolution than RYGB, concluding that OAGB is a technically easier procedure and features better glycemic control than RYGB.^29^ Li et al.,^30^ in a systematic review that encompassed 8 randomized trials, have reported that OAGB was associated with higher one-year excess weight loss, significantly fewer early post-operative complications, and shorter operative time compared to RYGB. Similarly, Uhe et al.^31^ in a systematic review that analyzed 25 randomized trials have reported that OAGB was associated with a 10% higher 1-year %EWL than RYGB, a finding comparable to that observed in the present study. Nevertheless, a consensus has been reached regarding the higher potential of OAGB to cause malnutrition because of its malabsorptive nature compared with RYGB.^32^ Thus, OAGB may lead to greater weight loss at the expense of more nutritional issues, which emphasizes the necessity of a rigorous postoperative multidisciplinary follow-up.

Younger age and worse baseline renal function were independent predictors of better postoperative renal outcomes in this study cohort. These findings are in accordance with previous evidence and, respectively, emphasize the importance of early surgical indication leading to better results, as well as the possibility of BS/metabolic surgery acting as a method to salvage individuals with already impaired renal function.^9-13^ It should be emphasized that this applies to individuals without established severe kidney dysfunction, considering that this study did not involve patients with end-stage renal disease. In fact, BS evidently plays a nephroprotective role through multiple mechanisms, such as the decrease in visceral fat volume and consequent reduction of chronic low-grade inflammation, the improvement of glycemic metabolism mediated by the activation of incretins, and antihypertensive effects associated with natriuretic properties of GLP-1 alongside weight loss itself.^33^ However, in individuals with kidney disease classified as stage 3 or worse already installed, the reversal rates after bariatric procedures are not significant, despite all other metabolic benefits.^34^

The long-term risk of biliary reflux-associated esophagogastric cancer after OAGB remains debatable. Recent studies by Keleidari et al.^35^ and Braga et al.^36^ have demonstrated that low rates of severe endoscopic and histopathological abnormalities were observed after 1 and 2 years after OAGB, respectively. One unique case of purely gastric cancer detected after OAGB was in the excluded stomach.^37^ The remaining cases were diagnosed at the esophagogastric junction, both 2 years postoperatively. Interestingly, neither patient had undergone biopsies of the esophagogastric junction, and one patient did not even undergo preoperative esophagogastroscopy.^38,39^ The commonly and historically described history of biliary reflux-associated cancer requires a significantly longer time of exposure, generally 20 years or more.^40,41^ Considering that OAGB has been systematically performed at least since 1997, no surge in the diagnosis of this type of cancer has been observed over recent years, as it would have been expected in case this operation really carried such risk.^42,43^ Nevertheless, continuous long-term endoscopic surveillance is warranted.

Considering the previously reported advantages of OAGB over RYGB, shorter operative time, lower perioperative morbidity, and greater weight loss and glycemic control,^44-47^ the current study demonstrates that OAGB is at least equivalent to RYGB in another significant postoperative outcome, which is the recovery of renal function.

This study had some limitations that should be considered. The small sample size of patients with OAGB and its short follow-up time are significant and should ensure the performance of larger prospective studies with longer postoperative follow-up periods. This decrease in serum creatinine levels may be related to surgically induced weight loss-related sarcopenia, at least to a certain extent. The GFR estimation model was appropriate for this population study model, although it cannot provide the same accuracy as direct measurements through total 24-hour urine collection and calculation of clearance, which are expensive and more difficult to execute. Moreover, changes in body composition postoperatively may have biased our findings. Meanwhile, the main strength of the current study was the systematic collection of renal function laboratory examinations after BS, which is not very common in most services.

## CONCLUSION

Compared to RYGB, OAGB led to an equivalent improvement in renal function 1 year postoperatively, along with higher weight loss.
